# Evaluation of Carum-loaded Niosomes on Breast Cancer Cells:Physicochemical Properties, In Vitro Cytotoxicity, Flow Cytometric, DNA Fragmentation and Cell Migration Assay

**DOI:** 10.1038/s41598-019-43755-w

**Published:** 2019-05-09

**Authors:** Mahmood Barani, Mohammad Mirzaei, Masoud Torkzadeh-Mahani, Mahboubeh Adeli-sardou

**Affiliations:** 1 Department of Chemistry, Shahid Bahonar University of Kerman, Kerman, Iran; 2 Young Researchers’ Society, Shahid Bahonar university of Kerman, Kerman, Iran; 3 Department of Biotechnology, Institute of Science, High Technology and Environmental Sciences, Graduate University of Advanced Technology, Kerman, Iran

**Keywords:** Electron microscopy, Breast cancer

## Abstract

Thymoquinone (TQ), a phytochemical compound found in Carum carvil seeds (C. carvil), has a lot of applications in medical especially cancer therapy. However, TQ has a hydrophobic nature, and because of that, its solubility, permeability and its bioavailability in biological mediums are poor. To diminish these drawbacks, we have designed a herbal carrier composed of Ergosterol (herbal lipid), Carum carvil extract (Carum) and nonionic surfactants for herbal cancer treatment. C. carvil was extracted and characterized by GC/Mass. Two different formulations containing TQ and Carum were encapsulated into niosomes (Nio/TQ and Nio/Carum, respectively) and their properties were compared together. Morphology, size, zeta potential, encapsulation efficiency (EE%), profile release rate, *in vitro* cytotoxicity, flow cytometric, DNA fragmentation and cell migration assay of formulations were evaluated. Results show that both loaded formulations have a spherical morphology, nanometric size and negative zeta potential. EE% of TQ and Carum loaded niosomes was about 92.32% ± 2.32 and 86.25% ± 1.85, respectively. Both loaded formulations provided a controlled release compared with free TQ. MTT assay showed that loaded niosomes have more anti-cancer activity compared with Free TQ and free Carum against MCF-7 cancer cell line and these results were confirmed by flow cytometric analysis. Cell cycle analysis showed G2/M arrest in TQ, Nio/TQ and Nio/Carum formulations. TQ, Nio/TQ and Nio/Carum decreased the migration of MCF7 cells remarkedly. These results show that the TQ and Carum loaded niosomes are novel carriers with high efficiency for encapsulation of low soluble phytochemicals and also would be favourable systems for breast cancer treatment.

## Introduction

Cancer has become a global deadly disease Such a way that about 7.6 million death reported in 2008 and based on anticipation, maybe it arrive to 13.1 million death in 2030^1^. Some traditional cancer therapies such as surgery and chemotherapy have some drawbacks that fail complete treatment of cancer. Also, chemical anticancer drugs can damage healthy cells because of high dose and prolonged usage^2^.

From the beginning and especially recent years, phytochemicals experienced an increasing demand for treatments of diseases. These compounds are bioactive that found in fruits, seed and other medical plants^3^. Doll and Peto were the first to point out an association between phytochemicals and cancer, suggesting that diets rich in vegetables and fruits reduce the risk of certain cancers^4^. Thomasset *et al*. (2006), in a review article, discussed dietary polyphenolic phytochemicals as an anticancer agent. They reported that some phytochemicals such as Genistein, Curcumin, Resveratrol and Epigallocatechin gallate (EGCG) have a promising anticancer effect on prostate and breast cancers^5^. In another review, Young-Joon Surh *et al*. (2003) described the mechanism and potential applications of dietary constituents on cancer therapy^6^. Also, Singh *et al*. (2017) performed Theoretical studies about inhibition activity of the androgen receptor (AR) of four phytochemical agents against prostate cancer. Their finding showed that Isobavachin have the best binding affinity (−13.73 kcal/mol)^7^. *Carum carvil* (C. carvil) is one of the best phytochemical compound that has anticancer potential also known as Persian cumin^8^. Some research showed that Carum carvil has an antioxidant effect that its antioxidant efficiency is better than Vitamin C^9^. Because of strong antioxidant activity, some researchers mentioned that it maybe has anticancer effect^10^. The main constituent of C. carvil is Thymoquinone (TQ) that exhibits high antioxidant effects^11,12^. TQ was found to be a good inhibitor in some cancer cell such as ovarian adenocarcinoma, uterine sarcoma, prostate cancer cells and cervical Cancer Cells. and hasn’t any toxic effect on non-neoplastic cells^13^. Rajput *et al*. (2013) evaluated apoptosis potential of TQ in breast cancer cells and showed that TQ promotes G1 arrest through translation inhibition of cyclin D1^14^. In similar study, Rajput *et al*. (2013) evaluated the apoptotic effects of TQ and Tamoxifen on XIAP mediated Akt regulation in breast cancer^15^. Another study that performed by Subhasis et (2012) proved that TQ and Diosgenin have an apoptotic potential in squamous cell carcinoma^16^. Despite remarkable success of chemoprevention by bioactive plant components in preclinical settings in animal models, the applicability in real life settings still has a long way to go. Some issues associated with these phytochemicals are low water solubility, poor bioavailability, high doses required, diverse genetic background of individuals at risk, etc^17^. Moreover, unsupervised concurrent administration of natural biomolecules and synthetic drugs may lead to drug failure due to unexpected severe side effects.

Nanotechnology is a promising field that has a lot of application in medicine. The use of nanotechnology in pharmaceutical filed called nanomedicine^18–20^. Encapsulation of food molecules into nanocarriers is being thoroughly investigated as a means to circumvent toxicity effects of synthetic drugs^21–23^. Indeed, nano-based formulations could enhance the efficiency of bioactive food components by improving their solubility and hence bioavailability. Additional benefits of nanoformulations are targeted delivery, controlled and sustained release, more interaction with selected cells, etc…^24,25^.

Niosomes (Nio) is an interesting type of colloidal nanocarriers that are made of cholesterol and non-ionic surfactants. Non-ionic surfactants have a hydrophilic head connected to a hydrophobic tail which doesn’t have any charge and is relatively non-toxic^26^. Generally, cholesterol served as a helper lipid that can diminish interactions of niosomes with proteins of the immune system and increase their stability in biological fluids^27^. Niosomes have a lot of unique properties versus liposomes such as chemically stability, long storage time, high bioavailability, low toxicity, accessible and low-cost raw materials and spontaneously loading of hydrophobic and hydrophilic agents^28,29^. Niosome can increase resistance and also the stability of active plant agent against degradation environment by encapsulating them and thus improving the bioavailability of phytochemical^30–35^. Because of hydrocarbon nature of phytochemicals, another critical goal for loading phytochemicals into niosomes is increasing their solubility in aqueous fluids. Rajput *et al*. (2015) reported novel multilamellar gold niosomes which were loaded with Akt-SiRNA and TQ and this nanoformulated delivery system had a promising activity against tamoxifen-resistant and Akt-overexpressing MCF7 breast cancer cells^36^. The targeted and controlled release of TQ and siRNA in MCF-7 cells enhanced the concentration of active agents in the active site and hence improved therapeutic efficacy than direct administration of therapeutic agents.

In this study, we evaluated the properties of TQ, TQ loaded niosome (Nio/TQ), Carum and Carum loaded niosome (Nio/Carum) and compared their efficiency on the breast cancer cell. The morphology, size, release behaviour and encapsulation efficiency of prepared niosomes were characterized. The *in vitro* cytotoxicity, flow cytometric analysis, DNA fragmentation and cell migration capacity of samples were also studied to investigate the anti-cancer activity. Results showed that this formulation has a good potential for encapsulating phytochemical compounds and also have promising anti-cancer activity. To the best of our knowledge, there is no information about encapsulating Carum into niosome and characterizing its anti-cancer properties.

## Results and Discussion

### GC-MS analysis

**Figure 1:**
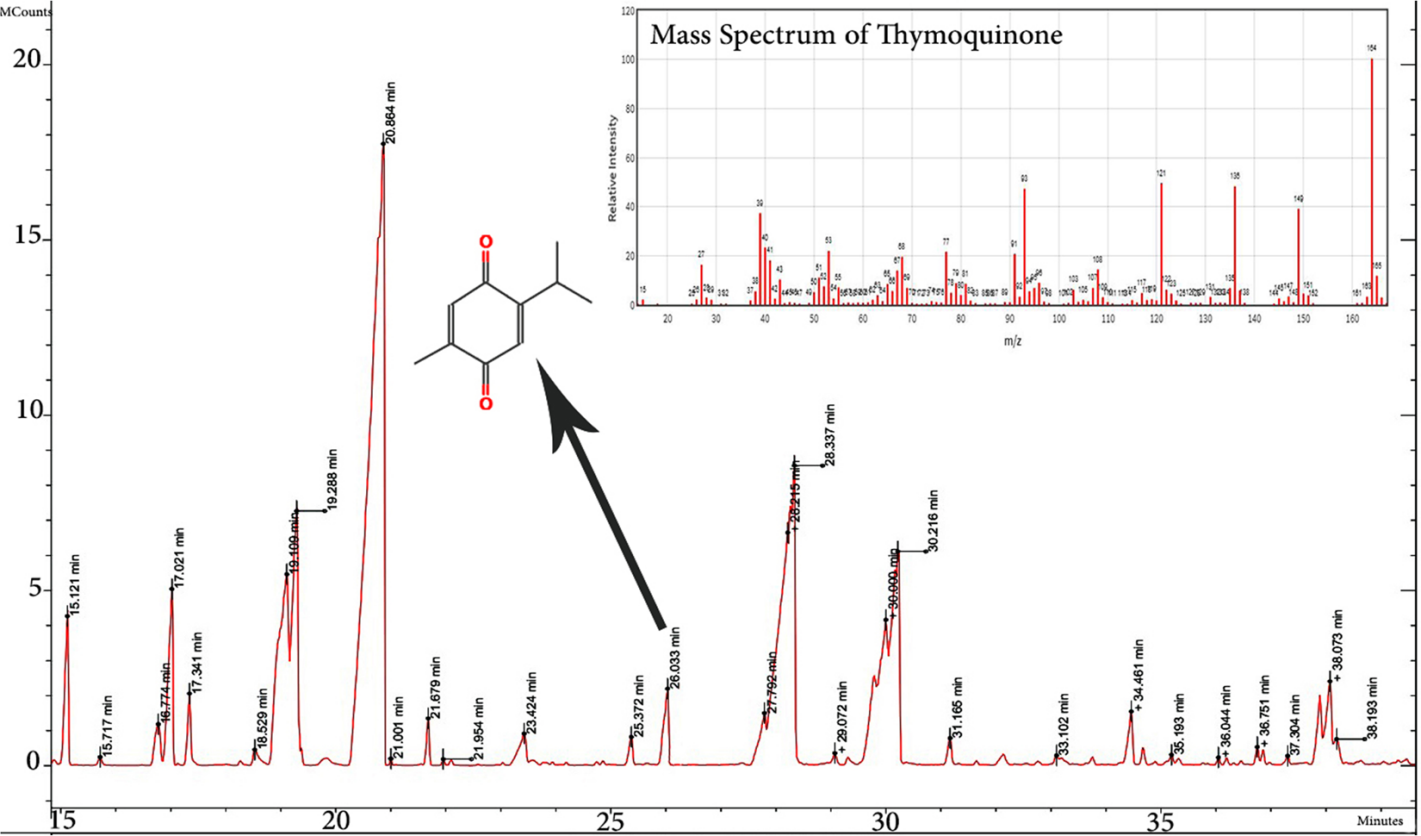
GC-MS chromatogram of Carum carvil extract (Carum) with the mass spectrum of Thymoquinone (TQ).

There are some beneficial compounds in C. carvil seeds, such as alkaloids, protein and essential oils. TQ is the most valuable compound of Carum carvil that about 406 articles focus on it since 1960. The GC-MS analysis of ethanolic extract revealed the presence of 13 components. The specific components of the essential oil and residue were identified by comparing mass spectra fragmentation pattern and retention time with the library values. As shown in Fig. 1, the amount of TQ was 2.21% that appeared in 26.03 minutes of retention time. Harzallah *et al*. (2011) evaluated the chemical composition of Tunisian Nigella sativa essential oil by GC–EIMS analysis^37^. They found that The significant components in the plant were p-cymene (49.48%), α-pinene (5.44%), β-pinene (4.31%) and γ-terpinene (3.69%), Whereas TQ represented only (0.79%). The amount of TQ in Carum was satisfactory high to show an anticancer effect against MCF-7 in comparison to standard TQ. Also, there are some other similar phytochemicals in Carum that maybe have the same function as TQ.

### Characterization of loaded niosomes

**Figure 2:**
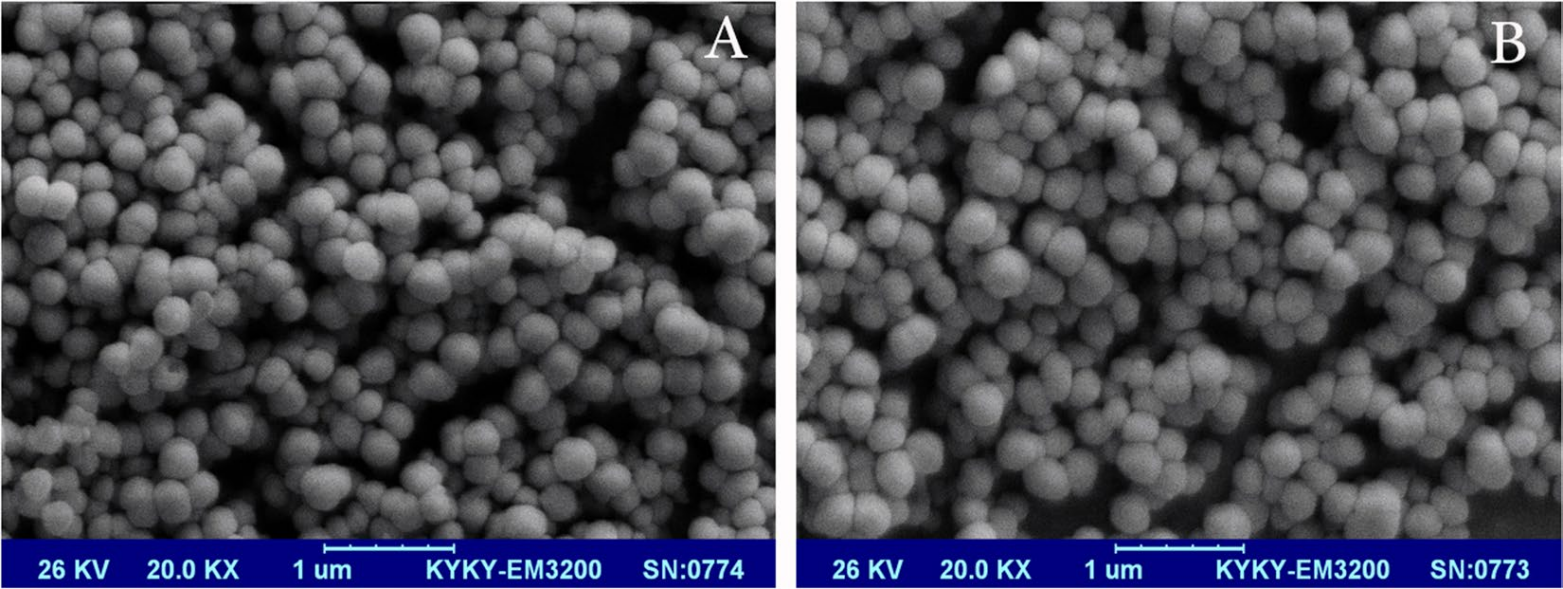
SEM images of Carum (**A**) and TQ (**B**)-loaded Niosome formulations, original magnification 20.000×.

One of the most important factors of a drug delivery system is stability and monodispresity and to prove that we have to characterize formulations by SEM and DLS analysis. SEM image showed that Carum (Fig. 2A) and TQ (Fig. 2B) loaded niosomes have a spherical morphology without any aggregation. As shown in Fig. 2, both formulations almost have a similar shape and size which confirms that in this case, loading of TQ and Carum don’t changed the morphology of formulations. The size of Carum and TQ loaded niosomes was in the range of 100–200 nm. Many authors mentioned that nanometric range (especially below 200 nm) of drug delivery system could guarantee successful arriving of nanocarrier to the tumour site^38^. Basiri *et al*. (2016) prepared α-Tocopherol-loaded niosome and evaluated its release behaviour. They reported that in the higher concentration of α-Tocopherol, the size of niosome formulation increased^39^.

**Figure 3:**
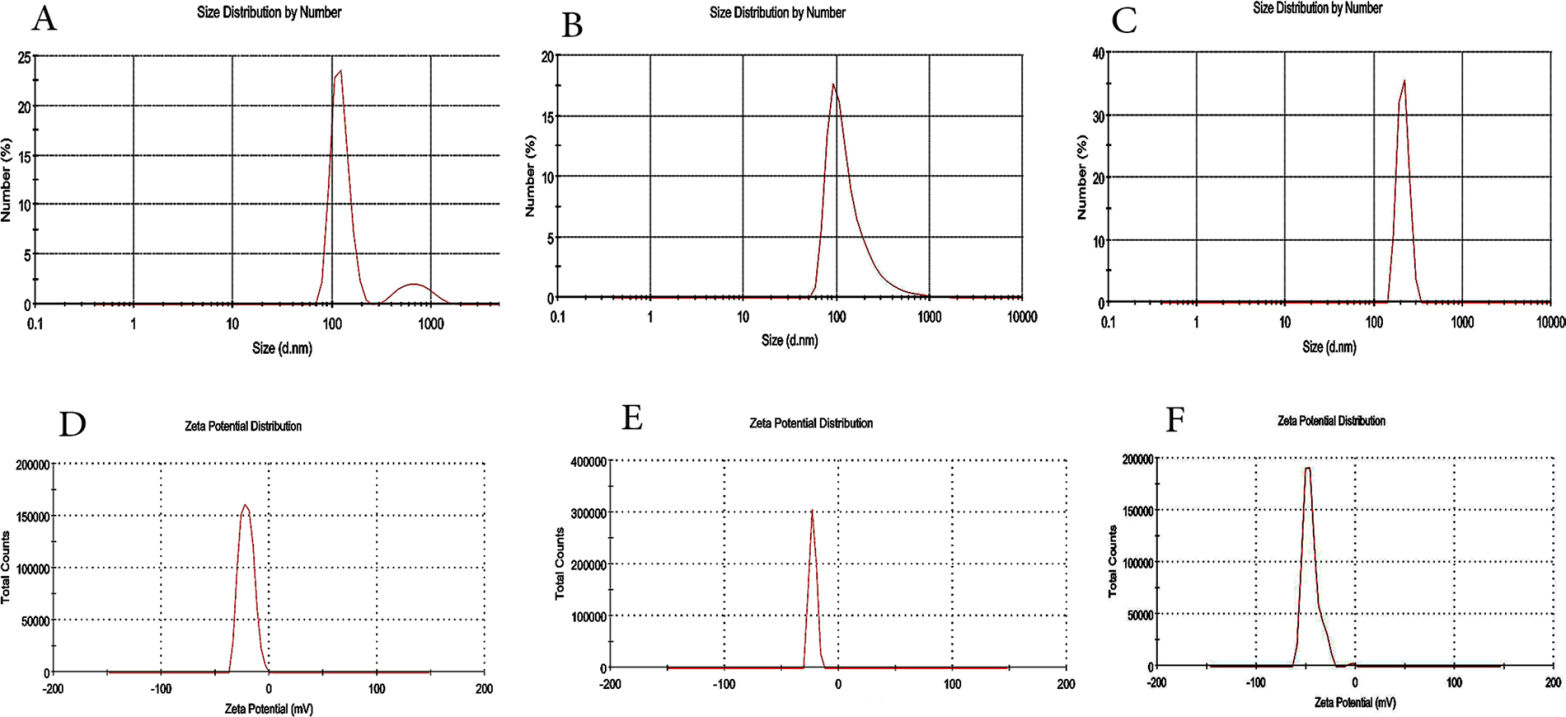
Size and Zeta potential of Bank niosome (**A,D**), TQ loaded niosome (**B,E**) and Carum loaded Niosomes (**C,F**) by DLS analysis.

DLS analysis was performed on blank niosomes (niosomes without TQ and Carum), Carum and TQ loaded Niosomes (Fig. 3). The homogeneity of the formulations can be evaluated by the PDI parameter. PDI ≤ 0.3 corresponds to an intense and small width peak in size distribution profile of particles^40^. The size of blank biosome was 122 nm with PDI value of 0.34 (Fig. 3A). Also sizes for TQ and Carum loaded niosomes were 102 ± 1.5 nm (PDI = 0.26) and 213 ± 2.1 nm (PDI = 0.21), respectively (Fig. 3A,B). Mashal *et al*. (2015) evaluated the size of prepared niosomes in the absence/presence of lycopene (DP60 and DP60L, respectively) and showed that incorporating of lycopene into niosome can increase the size of the resulted formulation^41^. However, the size of the niosomes is appropriate for tumor specific accumulation via EPR effect^42^. There is a good agreement size between SEM and DLS for TQ loaded niosome. A lot of authors mentioned that DLS shows hydrodynamic size of particles^43^. The hydrodynamic size measured by Dynamic Light Scattering (DLS) is defined as “the size of a hypothetical hard sphere that diffuses in the same fashion as that of the particle being measured”. Technically, in a particular suspension, particles are not absolutely spherical. So that, the particle size that calculated by DLS, is apparent size of particles that considered DLS theory and called Hydrodynamic diameter^44^. Since in Carum there are a lot of phytochemical compounds that can interact with surface of niosomes, we observed a bigger size in DLS than SEM for this formulation. Stability properties of emulsions are directly dependent on the Zeta potential values. Many authors mentioned that a zeta potential near ±30 mV could guarantee a long term stable formulation^45^. Zeta potentials for Blank niosome, Carum and TQ loaded niosomes were −19 mV, −22 mV and −41 mV, respectively (Fig. 3C,E,F). loading of phytochemicals into niosome resulted in more negative zeta values, lower PDI and hence more stable formulations.

Additionally, the Carum loaded niosome was kept at 4 °C for over six months, and less than 12 nm size increase was observed suggesting that this formulation have high stability over time (Data not shown).

One of the most critical needs of a nanocarrier is its potential to encapsulate drugs. An ideal nanocarrier should load higher content of drugs^46^. Drug Efficiency Encapsulation (EE%) for TQ and Carum was 92.32% ± 2.32 and 86.25% ± 1.85 indicating the successful loading of phytochemicals into Niosomes. Student t-test analysis was performed and showed that there is a significant difference between EE% of TQ and Carum loaded niosomes (p = 0.012). Because of hydrophobic nature of Carum and TQ, they incorporate in the bilayer of niosomes^28^. In a similar study, Thakkar & Brijesh (2017) synthesed niosomes containing curcumin and primaquine as an anti-malarial combination with a particle size of 220 nm and EE% of 82.12%^46^.

### Drug release behaviour of formulations

**Figure 4:**
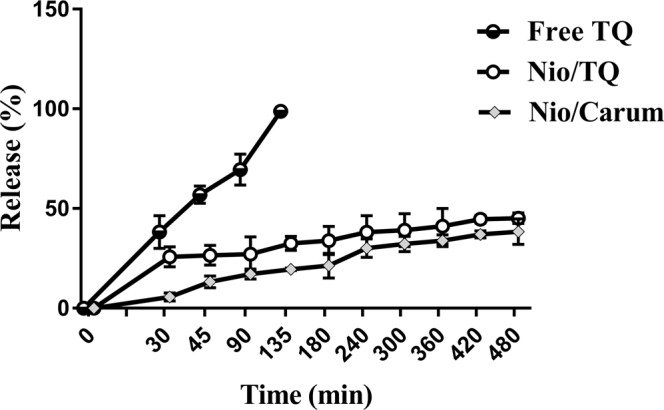
Release profile of TQ, TQ loaded niosome and Carum Loaded niosome in PBS 7.4 and 37 °C (n = 3).

Release rate performance is a required analysis for nanocarriers and give us an insight from the release behaviour of the drug in the body circulation^47^. The *in vitro* release profile of loaded formulations was investigated via dialysis method. The *in vitro* release behavior of loaded Niosomes and free TQ (TQ that not loaded into niosome) is shown in Fig. 4. A dialysis bag containing each formulation immersed in PBS solution (37 °C, pH 7.4). The kinetic release of drug in both loaded niosomes was biphasic. Release behaviour of drug showed a primary fast release and then a long term steady state. As shown in Fig. 4, the release rate of free TQ was very fast such a way that almost all of free TQ was released within 120 minutes. Interestingly, the release rates of TQ and Carum loaded niosomes were remarkably slower than free TQ. After 480 minutes, the release of TQ and Carum loaded niosomes was 45.15 ± 2.6% and 38.5 ± 6.3%, respectively. Thakkar *et al*. (2017) reported that hydrophobic materials that incorporated in bilayer part of niosome have a slower release rate than hydrophilic ones^46^. In this way, once intravenously administrated, the loaded niosomes could ensure minimal premature release of TQ during the circulation, and thereby greatly reduce required dose for effectiveness. Figure 4 is an approval for controlled and sustained release of TQ from niosome that is in agreement with other studies^48^.

### *In vitro* cytotoxicity assay

**Figure 5:**
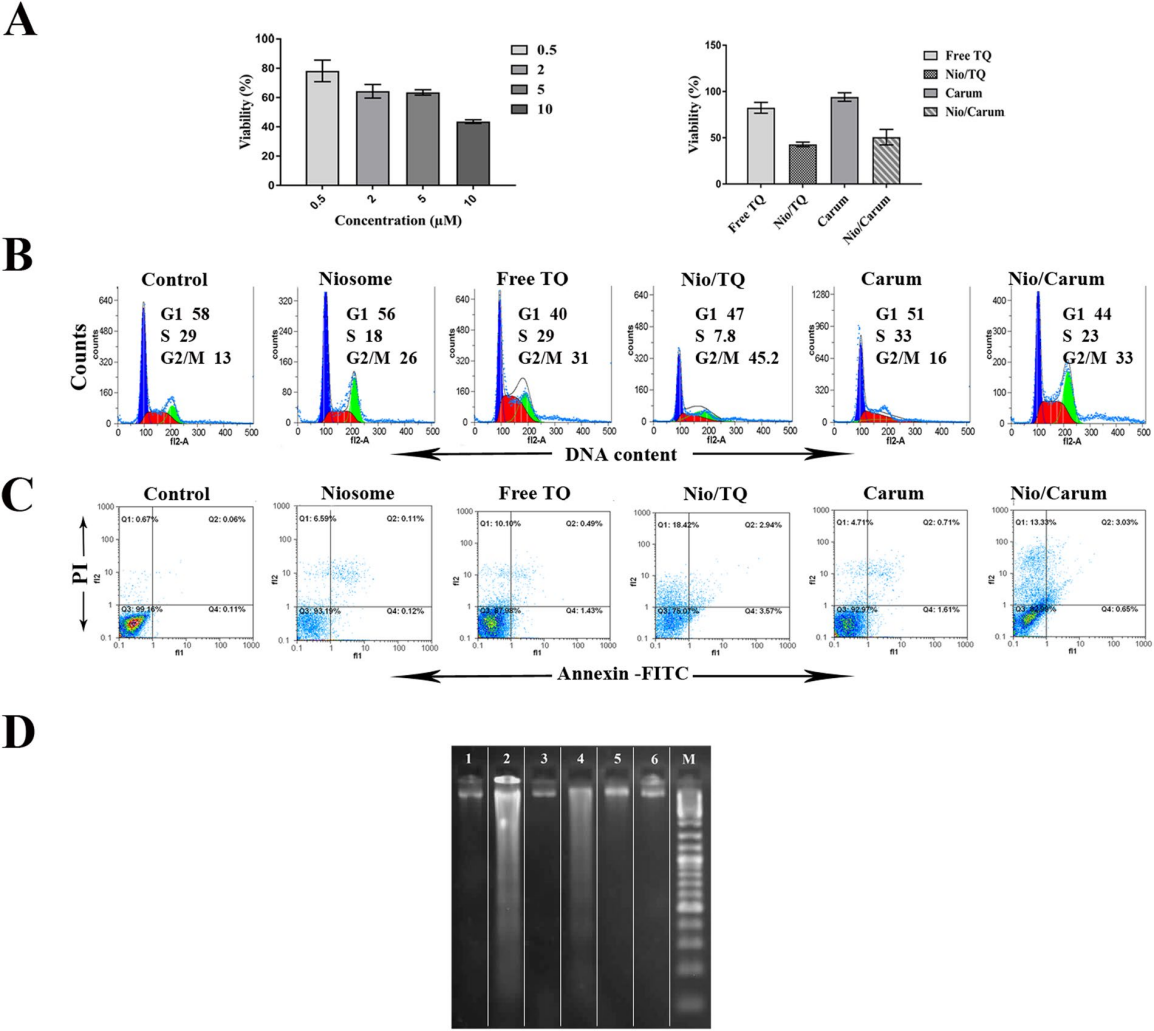
Cell viability assay of MCF-7 cells treated with 2 µM thymoquinone (TQ), TQ loaded noisomes (Nio/TQ), Carum extract (Carum) and Carum loaded niosomes (Nio/Carum(for 24 h. (**A**) MTT assay after treatment with different concentrations of blank noisome (0.5, 2, 5, and 10 µM) and MTT assay of free TQ, Nio/TQ, Carum and Nio/Carum. (**B**) Representative histogram plot of MCF-7 cells showing distribution in the different phases of the cell cycle after 24 h treatment, determined by flow cytometry after staining cells with PI. (**C**) Early, late apoptosis and necrosis of MCF-7 cells were analysed through flow cytometer after staining the cells with Annexin V-FITC and PI. (**D**) Detection of apoptotic DNA ladder in MCF-7 cell line by DNA fragmentation assay;(Lane M: Standard molecular size marker (1 Kb), Lane 1: free TQ, Lane 2: Nio/TQ, Lane 3: Carum, Lane 4: Nio/Carum, Lane 5: noisome and Lane 6: untreated cells The lanes are cropped from the same gel (a full image of the gel is provided in the Supplementary Information). Values were expressed as the mean of three different experiments ± SD.

The *in vitro* cytotoxic effect of the niosome formulations were tested using tetrazolium salt on MCF-7 cell line. Figure 5A shows viability of MCF-7 cells with blank niosomes at concentrations ranging from 0.5 to 10 µM for 24 h. Lower toxic effects were observed on cell growth for this formulation at 0.5 and 2 µM concentrations but decreased cell viabilities were observed as the concentration of niosome increased to 5 and 10 µM. At the amount of 2 µM, the percentages of viable cells 64.33%, but higher concentration of niosomes (≥2 µM) could result in higher cell toxicity of MCF-7 cells. Our results indicated that using of blank niosome at concentration of 2 µM has acceptable cytotoxicity for cells.

### Antitumor activity of loaded niosomes

The anti-tumor activity of TQ, Nio/TQ, Carum, Nio/Carum, balnk noisome and untreated samples was performed on MCF-7 cells in 2 µM concentation for 24 h. Figure 5A showed that TQ encapsulated in niosome has more antitumor activity than free TQ. As the cell viability of treated cells decreaced from 82.39 in TQ to 42.81 in Nio/TQ group. The viability of MCF-7 cells was also decreased from 94.06 in Carum to 50.68 in Nio/Carum groups. It has been reported previously, that combination therapy of TQ and tamoxifen, enhances the apoptotic and the growth inhibition of tamoxifen on MCF-7 cells and *in vivo*^15^. In another study, Rajput *et al*.^36^ reported TQ at a 40-μL/mL concentration exhibited 80% viability, whereas the same concentration of Nio-Au-TQ significantly reduced cell viability to 40%^36^. Here, there is a synergic effect that came from better internalizations of nano carrier and a controlled two phase release of TQ.

Cell cycle analysis of all samples showed a G2/M arrest in blank niosome, TQ, Nio/TQ, Carum and Nio/Carum formulations (Fig. 5B). Carum didn’t show any notable arrest against MCF-7 cells. Although cells were arrested in G2/M phase, former studies reported sub-G1 in MCF-7 cells treated with TQ and TQ/tamoxifen^15^. Rajput *et al*.^14^ explored that TQ promotes G1 arrest through translation inhibition of cyclin D1 and induces apoptosis in breast cancer cells. To evaluate whether activity of TQ, Carum and niosome containing these compounds is related to programmed cell death, the percentage of apoptotic and necrotic cells was measured in treated and untreated MCF-7 cells. As depicted in Fig. 5C, the apoptotic and necrotic activity of niosome formulations containing TQ and Carum was remarkably higher than free TQ and noisome formulations. loaded niosomes induces late apoptosis and necrosis. Carum didn’t show a notable apoptotic activity. DNA fragmentation assay also confirmed the apoptotic activity of Nio/TQ and Nio/Carum (Fig. 5D). Jun *et al*.^49^ demonstrated that TQ increases the apoptosis rate in both cervical cancer cells dose dependently. In summary, Fig. 5 confirmed that phytochemicals-loaded niosomes have a promising antitumour activity and can be used for a wide variety of active agents.

### Migration assay

**Figure 6:**
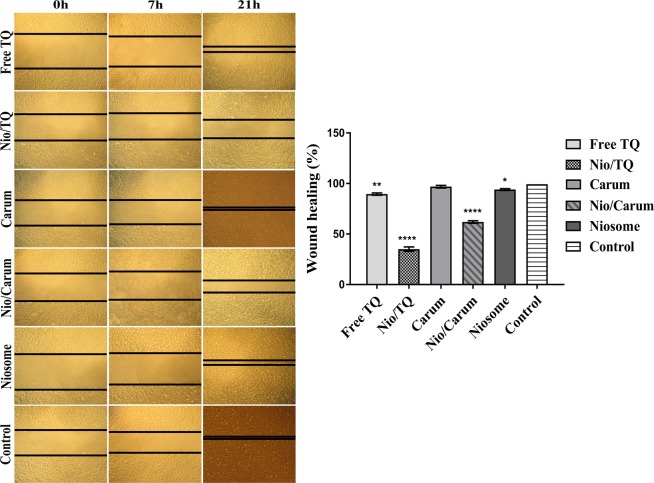
Effect of TQ, Nio/TQ, curum, Nio/carum and blank niosome on wound healing 0, 7 and 21 h after wound creation. The error bars represent the mean ± SD from three replicate experiments. *Represents *p < 0.05, **p < 0.01, ****p < 0.0001 was considered as significant.

Migration is one on the important characteristics of cancer cells and promote cancer metastasis. To determine the effect of TQ, Nio/TQ, Carum, Nio/Carum, balnk niosome and untreated samples on migration, *in vitro* wound (scratch) assays were performed in MCF-7 cells and the wound healing rate was monitored through the complete clousoure of the scratched. As shown in Fig. 6, TQ, Nio/TQ and Nio/TQ inhibited the MCF-7 cell migration remarkedly. In a similar study, the migration and invasion of TQ at a dose of 5 µM inhibited the growth, migration and invasion in both of CaSki and SiHa cell lines^49^.

## Conclusion

TQ despite the many advantages in cancer therapy, have a very low solubility in biological fluids that limits its applications. In this study, we introduced an innovative niosome delivery system with ergosterol (herbal lipid) in structure, instead of cholesterol which is usual in noisome synthesis, that encapsulated Carum and/or TQ and provided high stability, nanometric size, good encapsulation efficiency. Our findings suggest that Nio/TQ and Nio/Carum markedly inhibited the proliferation of breast cancer cells in comparison with free TQ and/or free Carum. Flow cytometric analysis and DNA fragmentation assay confirmed the apoptotic efficacy of these drug nanoformulated noisome systems. Furthermore, the migration of cancer cells was also suppressed in Nio/TQ and Nio/Carum formulations. This study suggests that encapsulation of low soluble phytochemicals such as TQ and Carum into niosomes can increases their effectiveness and hence can be considered as chemotherapeutic agents against breast cancer. Further studies should be performed to clarify the influence of other factors such as type of intracellular pathways, cellular uptake and *in vivo* study to better understand the molecular mechanism of these phytochemical carriers for the further development and establishment of Nio/TQ and Nio/Carum as clinical drugs.

## Material and Methods

### Materials

Sorbitane monostearate (Span 60), Polyoxyethylene sorbitan monostearate (Tween 60), MTT reagent, Ergosterol, dimethyl sulfoxide (DMSO) and standard Thymoquinone (2-isopropyl-5-methyl-1, 4-benzoquinone) was obtained from Sigma (St. Louis, MO, USA). Human breast cancer cell line (MCF7) was purchased from the National Cell Bank of Pasteur Institute, Iran. Dead Cell Apoptosis Kit with Annexin V FITC and PI for flow cytometry acquered from BioLegend, USA. SYBR-Safe DNA gel stain for gel electrophoresis was obtained from Invitrogen, USA.

### C. carvil extraction and GC/Mass analysis

C. carvil plant was received from Kerman, Iran. 50 gr of air-dried C. carvil was crushed, and extraction of the ethanolic solution was performed by Soxhlet. The resulted solution was concentrated by rotary evaporator at 180 rpm, 45 °C and two h^50^. The GC-mass instrument was a Varian 5300 gas chromatogram with a 2200 mass detector. The used column was a stationary phase HP-5 ms column with specifications of 60-meter length, 0.25 mm diameter and 0.25 µm thickness. Column oven program starts at 60 °C and hold for 5 min, then increased to 270 °C by a rate of 5 °C/min and hold in this temperature for 5 min. The flow rate of He as the carrier gas was kept at 1 ml/min. The temperature of the injection chamber was 260 °C with a split mode of 80:1. The mass spectrum obtained from the sample was compared with the database of the instrument (NIST-MS).

### Synthesis of loaded formulations

Thin film hydration technique or TFH was used for the synthesis of niosome formulations^51,52^. At the first, stock solutions of each niosome component at concantration of 50 mg/mL in choloroform was prepared. Then, Span 60, Tween 60, ergosterol and Carum or TQ at molar ratios of 30:30:30:10 were add to a RB flasks (50 mL). Rotary evaporator was used for cholorophorm evaporation (Eppendorf, Germany) at reduced pressure, 60 °C for 60 minutes. Resulted lipid film was hydrated with 5 mL of phosphate-buffered saline (PBS) 7.4 solution at 60 °C for 45 minutes. The obtained milky solution containing MLV niosomes was further sonicated for 10 min by Ultrasonic Bath Sonicator (Tecno-Gaz Ultrasonic system; Tecna S.p.A, Bologna, Italy) to produce SUVs that have a more homogenoius sizes. For further homogeneous formulation, filtration by membrane filter was performed (0.22 uM, Sartorius AG, Göttingen, Germany).

### Morphology of formulations by scanning electron spectroscopy (SEM)

Scanning electron spectroscopy technique (SEM) was used for morphology observation of formulations (SBC-12, KYKY, China). For this procedure, each niosome formolation were added to a double-sided carbon tape, positioned on an aluminium stub and vacuum dried. The samples were sputterd by Au at thickness of 200 nm by a Polaron E5100 for 3 minutes and Ar atmosphere. Then, images of each sample were taken at different magnifications.

### Size and zeta potential characterization of niosome formulations

Assessment of some critical parameters of formulations such as the mean hydrodynamic diameter, The particle size distribution, the polydispersity index (PDI) and zeta potential was performed by Dynamic Light Scattering technique or DLS. (Zetasizer Nano-ZS, Malvern Instruments Ltd., Worcestershire, UK). The DLS instrument had a power source of 4.0 mW and a He–Ne laser emitting at 633 nm. It was set for backscattering detection at a fixed scattering angle of 90°. Briefly, 2 mL of each Niosome formulation were diluted 1:20 and filtered with 0.45 um membrane filter (Sartorius AG, Göttingen, Germany) to avoid multiple scattering effects of sedimentations. Then, 2 mL of each sample poured in a polystyrene cuvette and analysed at 25 °C in triplicate to yield mean value.

### Determination of entrapment efficiency (EE%)

EE% of TQ was performed by UV-Visible spectrophotometer (Carry 100, Agilent). The spectrum was recorded between 750 and 190 nm to determine the characteristic peak of TQ. TQ at wavelenth of 330 nm had a characteristic peak. Calibration curve of TQ in 5, 10, 30, 50, 100 and 150 µg/mL concentration was recorded at 330 nm. For evaluation of EE% of loaded formulations, they centrifuged at 15100 rpm for 30 min and a temperature of 4 °C (5415D, Eppendorf, Germany). After that, supernatant resulted form centrifuge procedure was analyzed at 330 nm and EE% of TQ in the formulation was calculated as follow:

1 $$EE \% =\frac{({\rm{Total}}\,{\rm{amount}}\,{\rm{of}}\,{\rm{TQ}}-{\rm{TQ}}\,{\rm{in}}\,{\rm{supernatant}})}{{\rm{Total}}\,{\rm{amount}}\,{\rm{of}}\,{\rm{TQ}}}\times 100$$

### Release experiments

First, one mL of each loaded formulation was added to a dialysis bag (molecular weight cut off 12,000 Da) and clipped from two ends and placed in a beaker containig 50 mL PBS 7.4 as a release medium. The beaker was stirred at a constant rate of 150 rpm by a magnetic stirrer at 37 °C for eight hours (Heidolph, MR300 µG, Germany). At each specific time point, One mL of the released content of dialysis bag to the PBS (release medium) was taken for UV-Vis analysis (at a wavelength of 330 nm) and instantly replaced with new PBS buffer. The percentage of released TQ from niosome presented as release profile. Profiles of release rate were drawn by Graph Pad Prism software (version 6, San Diego, CA, USA).

### Cell culture study

The MCF7 cells were cultured in RPMI1640 medium (Invitrogen, USA) supplemented with fetal bovine serum (FBS) 10% (v/v) and antibiotics (penicillin 0.1 μg/μL and streptomycin 0.1 μg/μL) in a humidified incubator with an environment of 95% air and 5% CO_2_ at 37 °C. The culture medium was refreshed every two days.

### Cytotoxicity assay

*In vitro* cell cytotoxic effect (MTT) of TQ, Nio/TQ, Carum extract, Nio/Carum, at the final concentration of 2 µM, and different concentrations of blank niosomes (0.5, 2, 5 and 10 μM) on MCF-7. Briefly, the MCF-7 cells were seeded into 96-well plates at 7 × 10^3^ cells per well and incubated for 24 h. then, the incubated cells were treated with mentioned compounds and encubated for 24 h. After the incubation time, 100 mL sterile MTT solution (0.5 mg/mL) was added to the wells of plate and incubated again for 4 hr at 37 °C. finally, 100 μL of DMSO solvent was added. In the end, the absorbance was measured at 570 nm^53^.

### Flow cytometric analysis

To investigate the effect of TQ, Nio/TQ, Carum, Nio/Carum and blank niosome formulation, MCF7 cells were treated with two μM of compounds at 37 °C for 24 h. After treatment, the cells were trypsinized by Trypsin-EDTA treatment and washed with PBS. Then cells were stained with 50 μg/mL PI solution containing 0.1% Triton X-100 and 0.1% sodium citrate^54^. The distribution of cells in various cell-cycle phases was analyzed by flow cytometer (Partec, Germany) and Flow max 2.3 software.

To study the apoptosis rate, the treated cells were harvested and stained with Annexin V-FITC with PI for 15 min under dark condition, according to the manufacturer’s protocol (BioLegend, USA). The apoptotic cells were then detected on the flow cytometry, and representative histograms of apoptotic versus live cells and necrotic cells were generated.

### DNA fragmentation study

For DNA fragmentation assay was performed based previous studies^55,56^ with some modifications. Briefly, 2 × 105 cells were treated with TQ, Nio/TQ, Carum, Nio/Carum and blank niosome (with the final concentration of 2 μM) for 24 h. Because of the floating apoptosis cells, the media of cultures was collected and centrifuged at 5000 rpm for 5 minutes. The treated and control negative cells were trypsinized, harvested and washed three times with PBS. The cells were then lysed with 500 μL of lysis buffer (20 mM Tris-HCl (pH 8), 10 mM EDTA (pH 8) and 0.5% Triton X-100). After a 30-min incubation on the ice bath, the lysates were centrifuged at 10,000 × g for 20 min. RNase A was added to the supernatant A at the final concentration of 100 μg/mL at 37 °C for one h.

The DNA was extracted with a similar volume of phenol/chloroform for 10 min at 25 °C and next centrifuged at 10,000 × g for 5 min. The upper phase was mixed with an equal volume of cold isopropanol and incubated at −20 °C overnight. DNA was separated by centrifugation at 10,000 × g for 10 min. The pellet was air-dried for 10 min at room temperature and dissolved in distilled water. The DNA samples were electrophoresed on a 2% agarose gel containing 100 μL/100 mL SYBR-Safe DNA gel stain (Invitrogen, USA) and photographed by an ultraviolet gel documentation system (Bio-Rad, USA).

### Migration assay

To determine the effect of all tested compounds on MCF7 cell migration, the cells were seeded on 12-well plates in RPMI 1640 growth medium to reach 90–95% confluency. The next day, similar wound area was introduced to monolayer cells by a sterile yellow pipette tip, and TQ, Nio/TQ, Carum, Nio/Carum and blank niosome in the final concentration of 2 µM (in culture medium) were added to each well. The scratched area without any treatment considered as control. The speed of wound closure was assessed by measuring the scratched area at different time intervals (0, 7 and 21 h). The ImageJ software (NIH, Bethesda, MD, USA) was used to quantify the percentage of cell migration^49^.

### Data-analysis

All experiments were achieved at three times and values are represented as mean ± SD. All data investigated by One-way ANOVA method. Also a p-value < 0.05 was considered as a significant difference.

## Supplementary information

Supplementary
