# Supplementary material for: Evaluation of Carum-loaded Niosomes on Breast Cancer Cells:Physicochemical Properties, In Vitro Cytotoxicity, Flow Cytometric, DNA Fragmentation and Cell Migration Assay
Source: Sci Rep. 2019 May 9;9:7139. doi: 10.1038/s41598-019-43755-w (PMC6509162; doi:10.1038/s41598-019-43755-w)

**Evaluation of Carum-loaded niosomes on Breast Cancer Cells:Physicochemical Properties, In Vitro Cytotoxicity, Flow Cytometric, DNA Fragmentation and Cell Migration Assay**

*Mahmood Barani^a,b^, Mohammad Mirzaei^a^, Masoud Torkzadeh-Mahani* ^c^,* *Mahboubeh Adeli-sardou^c^*

*^a^Department of Chemistry, Shahid Bahonar University of Kerman, Kerman, Iran.*

*^b^Young Researchers’ Society, Shahid Bahonar university of Kerman, Kerman, Iran.*

*^c^Department of Biotechnology, Institute of Science, High Technology and Environmental Sciences, Graduate University of Advanced Technology, Kerman, Iran.*

**masoud.torkzadehmahani@gmail.com*

*Tel: +98-3433776611, Fax: +98-3433776617*


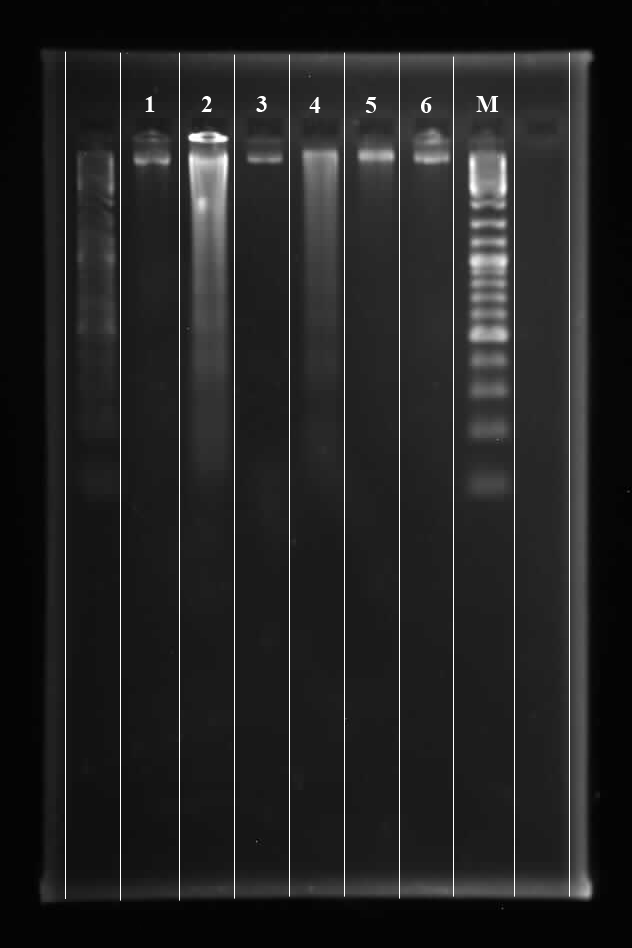

Supplement: Supplementary file 1 — Supplementary [file 41598_2019_43755_MOESM1_ESM.docx]
